# Enhanced YOLOv8 Ship Detection Empower Unmanned Surface Vehicles for Advanced Maritime Surveillance

**DOI:** 10.3390/jimaging10120303

**Published:** 2024-11-24

**Authors:** Abdelilah Haijoub, Anas Hatim, Antonio Guerrero-Gonzalez, Mounir Arioua, Khalid Chougdali

**Affiliations:** 1 Engineering Sciences Laboratory, National School of Applied Sciences of Kenitra, Ibn Tofail University, Kenitra 14000, Morocco; 2 Laboratory of Research on Sustainable and Innovative Technologies (LaRTID), National School of Applied Sciences of Marrakech, Cadi Ayyad University, Marrakech 40000, Morocco; 3 Department of Automation, Electrical Engineering and Electronic Technology, Universidad Politécnica de Cartagena, 30202 Cartagena, Spain

**Keywords:** maritime surveillance, unmanned surface vehicles, YOLOV8 enhancement, edge devices, energy efficiency, accuracy, machine learning

## Abstract

The evolution of maritime surveillance is significantly marked by the incorporation of Artificial Intelligence and machine learning into Unmanned Surface Vehicles (USVs). This paper presents an AI approach for detecting and tracking unmanned surface vehicles, specifically leveraging an enhanced version of YOLOv8, fine-tuned for maritime surveillance needs. Deployed on the NVIDIA Jetson TX2 platform, the system features an innovative architecture and perception module optimized for real-time operations and energy efficiency. Demonstrating superior detection accuracy with a mean Average Precision (mAP) of 0.99 and achieving an operational speed of 17.99 FPS, all while maintaining energy consumption at just 5.61 joules. The remarkable balance between accuracy, processing speed, and energy efficiency underscores the potential of this system to significantly advance maritime safety, security, and environmental monitoring.

## 1. Introduction

The increasing importance of controlling and managing the maritime domain, which encompasses both coasts and the open sea, is undeniable for a wide array of civil and military applications. Ensuring the safety, security, and environmental stewardship of these vast areas demands robust monitoring and control systems. Traditionally, the management of such a domain has relied heavily on manual efforts, requiring substantial manpower endowed with specialized skills, a method that is both complex and challenging. Enter the Unmanned Surface Vehicles, a revolutionary step forward in the domain of maritime management. USVs empowered by Artificial Intelligence, present a significant leap towards automating and enhancing maritime domain monitoring tasks. These autonomous systems are engineered to perform a multitude of functions ranging from pollutant spill detection and maritime traffic monitoring to the management of fishing operations and the identification and tracking of vessels [1].

Deploying AI in maritime environments presents challenges; however, USVs equipped with AI and video surveillance navigate these environments with exceptional precision. Studies highlight USVs’ versatility, including environmental preservation [2], navigating difficult terrains [3], and charting in shallow waters [4]. Further research [5,6] has delved into their roles in various maritime missions and environmental monitoring, underscoring USVs’ technological advancements and their importance for maritime management and conservation. Our focus is on enhancing maritime surveillance, aiming to boost security and monitoring through advanced object detection techniques. We explore three methodologies: image processing, machine learning, or their combination on edge devices, to significantly improve maritime object recognition. Advances in image processing are significantly enhancing maritime surveillance. The integration of spatial–temporal algorithms and systems for detecting marine objects from UAV footage, as discussed in [7,8], demonstrates significant progress in identifying floating objects without presupposing specific maritime conditions. Further elaborating on detection capabilities, refs. [9,10] illustrated how leveraging low-level features, edge-based segmentation, and Histogram of Oriented Gradient HOG methods surpass traditional object detection approaches. Moving towards practical applications, ref. [11] unveiled a computer vision technique for autonomously identifying marine vehicles through buoy camera systems. Complementing these advancements, ref. [12] presented a real-time semantic segmentation model for USVs, streamlining obstacle mapping. In the realm of vessel detection, refs. [13,14] explored efficient algorithms for analyzing both aerial and infrared sea-sky imagery, emphasizing their flexibility under diverse environmental conditions. Comprehensively, ref. [15] brought to light a multi-sensor surveillance system adept at tracking a variety of vessels, effectively adapting to changing visual environments. On another note, machine-learning-based detection models have brought significant advancements in maritime surveillance. Techniques like transfer learning on pre-trained CNNs have been instrumental, with studies like [16] achieving remarkable ship classification accuracy. The versatility of CNNs in identifying ships using both real and simulated data are further demonstrated by [17,18]. Innovations continued with [19]’s enhanced Faster R-CNN and [20]’s feature structure fusion method improving detection efficiency and classification accuracy. Moreover, ref. [21] introduced an optimized regressive deep CNN, showcasing high accuracy in ship detection and classification for intelligent maritime management.

On another front, integrating machine learning with image processing techniques has significantly refined maritime surveillance capabilities. Techniques combining SVM classification and novel feature extraction, as seen in [22,23], markedly advance ship detection accuracy. Utilizing DCT-based algorithms, [24] demonstrated efficiency on dynamic sea surfaces, streamlining detection on non-stationary platforms. The S-CNN model [25] optimized high-resolution image analysis for diverse ship detection, complemented by saliency-aware approaches in [26,27,28] that refine detection in complex backgrounds. Additionally, ref. [29]’s ensemble framework targets ship detection in coastal videos, showcasing improved accuracy across various maritime conditions. Advances in ship detection leverage both single-stage and two-stage neural networks, with two-stage models like Mask R-CNN and Faster-RCNN offering high accuracy. These models, enhanced through self-supervised learning and attention mechanisms, improve fine segmentation, which is crucial for maritime surveillance [30,31]. Such approaches achieve high precision even in complex maritime environments [32]. With the rapid evolution of YOLO models as a single-stage approach, especially in maritime surveillance, significant strides have been made towards enhancing detection precision and real-time performance. Innovations like the enhanced YOLO v3 tiny [33] and Light-YOLOv4 [34] prioritize speed and accuracy, optimizing for edge device deployment. Integrations such as CBAM with YOLOv4 [35] and the exploration of YOLO versions on embedded devices [36] push the boundaries of efficiency. The lightweight, YOLO-based detector LMSD-YOLO [37] addresses multi-scale target detection challenges, emphasizing the need for compact, high-performance models. Further advancements are seen in dataset refinement, with the corrected Singapore Maritime Dataset-Plus [38], and the application of YOLOv7 [39] and eYOLOv3 [40] for ship detection under different weather conditions, underscoring the continuous improvement in maritime object detection technologies.Transitioning to object tracking in our perception system concept, recent advancements underscore the role of automated tracking in enhancing maritime safety and surveillance. Techniques such as robust real-time ship tracking [41], which mitigates the impact of sea waves through advanced background subtraction, and multi-spectral tracking [42], which highlights IR data’s effectiveness at seaport entrances, are pivotal. Moreover, ref. [1] highlighted the significance of deploying autonomous surface vehicles (ASVs) equipped with AI-based image recognition services for monitoring marine protected areas (MPAs). Additionally, using video surveillance-based methods [43], ref. [44] showcased their utility across diverse maritime settings, adapting to various lighting and weather conditions. These efforts emphasize the importance of combining machine learning and video analytics in maritime surveillance, while also showcasing ongoing innovations in automatic detection and tracking systems to improve maritime safety.

This article’s main contributions are the following: it introduces an innovative architecture and perception system specifically designed for an Unmanned Surface Vehicle (USV) focused on tracking, detailing aspects such as its physical structure, operational methods, communication strategy, and energy efficiency. Additionally, it presents the deployment of a specialized detection and tracking model on the NVIDIA Jetson TX2, an edge computing device selected for its ability to meet the real-time maritime surveillance demands of the USV system, showcasing the integration of advanced technology to boost performance. Finally, the article demonstrates the successful implementation of the model on the Jetson TX2, emphasizing its effectiveness in real-time detection and tracking, which enhances autonomous navigation and surveillance in marine environments.

The article is organized as follows. Section 2 discusses methodology, covering USV architecture, communication, target selection, energy management, and perception module optimization. Section 3 details hardware and software implementation, performance metrics, and dataset. Section 4 presents results and discussion, including comparisons with existing studies. Section 5 concludes the article.

## 2. Methodology

In this section, we lay out the methodology used to develop, integrate, and evaluate a cutting-edge maritime surveillance system utilizing an Unmanned Surface Vehicle. Our approach prioritizes efficient data flow management and aims for high accuracy in detecting and tracking marine targets. We also focus on ensuring effective communication with the Ground Control Station, especially for choosing targets to track, showcasing our commitment to maximizing resource use and operational performance of the USV. Our quest for efficiency led us to select and compare different versions of the YOLO object detection model, with special attention to YOLOv8 [45] for its significant advantages in accuracy and efficiency. While promising for enhancing detection precision, integrating the CBAM module [46] increased complexity and energy consumption. This led us to explore various configurations of modules like the Convolution (Conv) module, Depthwise Convolution (DWConv) [47], Cross Stage Partial with two convolutions (c2f) [48], C3 Ghost (C3Ghost) [49], and Bottleneck CSP (BottleneckCSP) [48] to find the ideal balance between detection accuracy and energy efficiency. Moreover, incorporating centroid tracking into the perception module establishes an economical method for accurately tracking detected marine targets. A significant portion of our methodological approach is dedicated to optimizing the system’s energy consumption. This focus on energy management, detailed towards this introduction’s end, underscores the importance of developing a USV system that is not only effective in surveillance and tracking but also sustainable in terms of energy efficiency.

The methodology involves several key modules, each contributing to the comprehensive functionality of our USV system. We start with the module that ensures communication and target selection, the backbone of real-time command and data relay between the USV and its control center.

### 2.1. Communication and Target Selection

The Communication and Target Selection module is essential for enabling seamless and reliable data transmission between the Ground Control Station (GCS) and the Unmanned Surface Vehicle (USV). This module ensures that the USV can receive commands and send telemetry data, supporting effective mission control and situational awareness. The architecture facilitates the exchange of information necessary for real-time operations and decision making, forming the backbone of the USV’s interaction with its control systems.

In this section, we explore our proposed architecture for communication between the Ground Control Station (GCS) and the Unmanned Surface Vehicle, as well as between the USV and the onboard computer. Under normal circumstances, the USV operates in a mode selected by the pilot, which falls within the predefined modes of the autopilot responsible for managing USV actions without intervention from the onboard computer, which remains in sleep mode. Commands are transmitted by the pilot via RF communication (serial port) using Ground Data Terminal (GDT) and data terminal installed on the USV. These commands can originate from a Remote controller or a Ground Station Computer, where an application that manages communication with the autopilot, like Mission Planner for Pixhawk and PX4, is installed. This application can configure the autopilot according to the USV’s needs, plan missions, change modes, and receive and display telemetry data for monitoring our USV (GPS, IMU, heading, motor status, chosen mode, etc.). Surveillance camera frames are sent via the same Ethernet datalink port and displayed in our application at the GCS, covering the communication between the GCS and autopilot under normal operations.

Once the pilot decides to switch to intelligent surveillance mode, they activate the detection and identification mode through the camera application. A frame is sent via the Ethernet port over TCP/IP through the datalink to the USV, received by the onboard computer connected to the datalink. The onboard computer then receives the camera payload images, beginning detection and identification (details of which will be discussed in the following section). The results of this processing are sent via the Ethernet port back to the GCS and displayed in the surveillance camera application. When the pilot chooses a target, a frame is sent to the onboard computer via Ethernet. The Jetson TX2 receives this frame, stops detection, and begins tracking (discussed in detail in the next section), communicating with the autopilot to change the current mode to guided mode. The tracking result serves as input to the control block responsible for keeping the target centered in the camera’s view with smooth regulation. Control outputs are sent to the autopilot via serial communication using the MAVLink protocol, and USV feedback is retrieved from the autopilot via MAVLink (in our case, Robot Operating System (ROS) is used with MAVROS to communicate with autopilots that support MAVLink). The onboard computer takes command of the USV in tracking mode until it receives a frame from the pilot to stop or switch back to detection and identification mode. If it stops, a command is sent to the autopilot to switch back to the previous mode and the onboard computer is switched to sleep mode. If detection is enabled, tracking is stopped and detection and identification are initiated. The choice to activate/deactivate tracking and disable/enable detection and identification, detailed in the power management section, highlights our comprehensive approach to managing both the operational and energy efficiency of our surveillance system.

**Figure 1:**
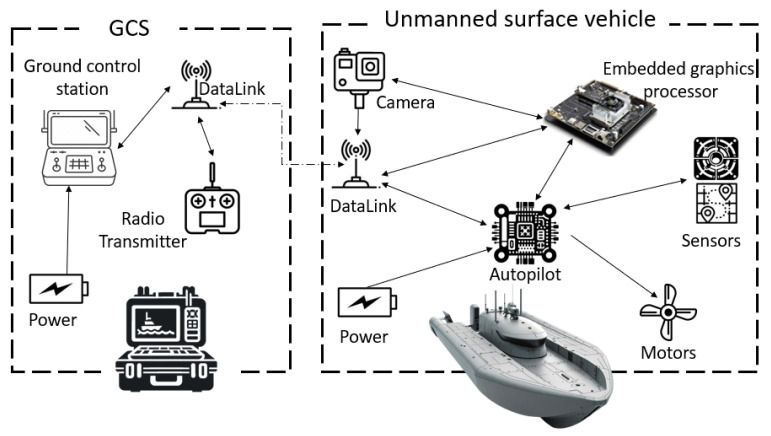
Physical configuration of the USV and GCS components.

**Figure 2:**
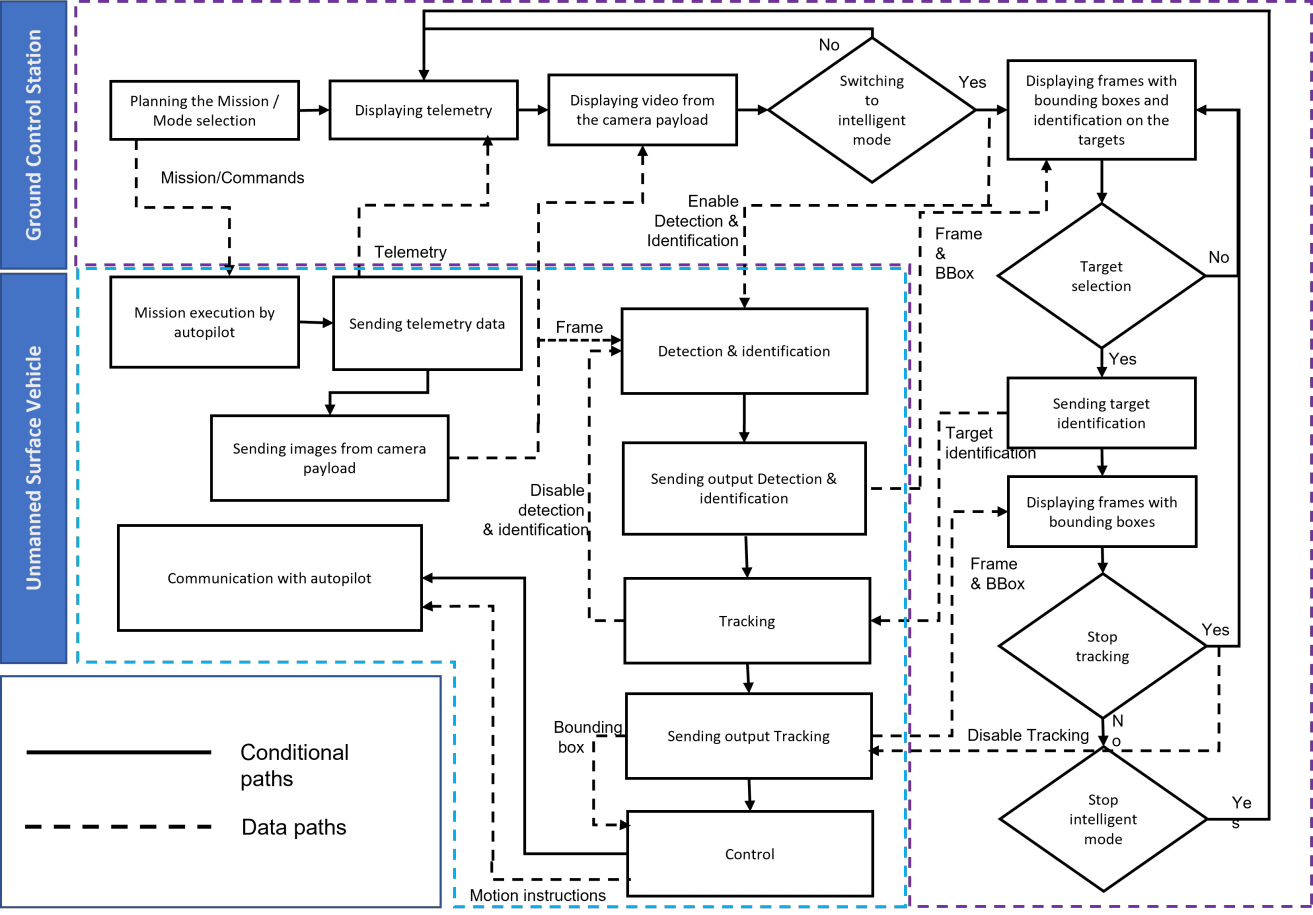
Architecture and operational flow of USV system.

To illustrate our architecture, Figure 1 offers a visual representation of the physical arrangement of components within the USV and the GCS, emphasizing the integration of each element into the overall system. The diagram in Figure 2 further elaborates on the communication architecture, data flows, and functional operations outlined previously, providing a clear visual guide to comprehend the complex operations of our USV design.

With the communication and target selection system ensuring seamless data transmission and operational control, the next important step is the USV’s perception module, responsible for detecting and tracking maritime objects.

### 2.2. Perception Module and Optimization

In this section, we detail the development of our advanced perception system for the Unmanned Surface Vehicle, aimed at enhancing its capabilities for maritime missions through a detection and tracking system specifically designed for embedded devices like the Jetson TX2. The perception module serves as the core of the USV’s visual processing capabilities, enabling it to identify and track objects accurately in real-time. This module integrates various subcomponents that optimize the balance between accuracy and energy efficiency, crucial for sustained maritime operations. Our exploration with YOLOv8 led to targeted architectural adjustments to minimize energy consumption while maximizing performance, ensuring the model’s suitability for our platform. Although the idea of integrating attention modules such as CBAM was initially considered to increase detection accuracy, their impact on computational load directed us towards alternative optimizations. This resulted in a continuous improvement process for our perception system, seeking a balance between real-time performance and energy efficiency.

To highlight YOLOv8’s advantages over previous versions, we conducted a comparative analysis with YOLOv4 and YOLOv5. Our results demonstrated that YOLOv8 achieved an mAP0.5 of 0.99075 and an FPS of 17.95 with an energy consumption of 5.62 joules on the Jetson TX2 at a resolution of 640 × 640. In contrast, YOLOv4 Tiny [50] reached an mAP0.5 of 0.89 and an FPS of 82 on the Jetson Xavier NX, while YOLOv5x pruned [51] recorded an mAP0.5 of 0.963 and an FPS of 19.09 on the same platform but at a lower resolution of 204 × 204. These findings underscore YOLOv8’s superior balance of accuracy, energy efficiency, and real-time processing capabilities, making it an optimal choice for energy-constrained, real-time maritime surveillance tasks. Detailed analysis and further discussion of these results are provided in the Section 4.

In the following, we will detail every component of our perception system, delving into the intricacies that define its functionality and effectiveness.

#### 2.2.1. Detection Component

The Detection Component is a fundamental part of the perception system that enables the USV to identify and respond to objects within its operational environment. This module is vital for effective maritime surveillance, where accurate and efficient detection is essential for safe and autonomous navigation. Ensuring a balance between detection performance, speed, and energy efficiency is important for maintaining the USV’s operational autonomy over extended missions.

Ship detection in maritime environments is a critical challenge in the field of navigation and maritime surveillance, particularly for solutions involving Unmanned Surface Vehicles. In this context, the effectiveness of detection is not the only performance criterion to consider. Execution speed, appropriate accuracy, and low energy consumption are key factors that help maintain the USVs’ energy autonomy, essential for extended missions at sea. While numerous methods and models exist for maritime detection, as discussed in the related work section, the recent trend involves integrating advanced detection models with specific enhancements. According to [40], which conducted experiments on the same dataset and used graphic cards similar to our target, the Nvidia Jetson TX2 and the Nvidia Jetson Xavier, it appears that YOLOv7 Tiny offers the best performance. However, this finding prompted us to further explore the latest architecture, YOLOv8. In our pursuit of improvement, we attempted to integrate transformers, specifically CBAM, into YOLOv8. Our tests of various architectures incorporating CBAM revealed models with computational complexity ranging from 11.8 to 19.6 GFLOPS, which is higher than YOLOv8 without modifications. Despite the integration of CBAM, the increase in accuracy proved to be modest, and energy consumption increased due to the model’s increased complexity. In this segment, we will focus on the architecture of YOLOv8 and the enhancements we have made. We will detail our proposed architecture, highlighting our strategy for balancing performance, accuracy, and energy efficiency. This approach aims at optimizing the detection system on USVs, offering a viable and efficient solution for autonomous maritime surveillance.

The YOLOv8 architecture [52] demonstrates a sophisticated design, tailored to excel in object detection tasks through a harmonized combination of its backbone, neck, and head components. Each segment plays a key role in processing and interpreting the visual data, allowing for precise and efficient detection outcomes. Here is an exploration of these components:

- Backbone: The backbone of YOLOv8, essential for extracting features, starts with layers of convolutions that deepen channels while narrowing the input’s spatial size. Designed for precise feature extraction from various scales, it progresses from smaller (64, 128) to larger (up to 1024) filters, each enhanced by a C2f (BottleneckCSP with two convolutions) module for improved integration. Additionally, an SPPF (Spatial Pyramid Pooling-Fast) layer pools contextual details across scales, ensuring the features are detailed and reflective of the inputs.
- Neck: The neck connects the backbone to the head, functioning as an intermediary that refines and restructures the features extracted by the backbone for optimal detection performance. It employs a series of upsampling and concatenation operations to merge features from different levels of the backbone, allowing the model to maintain spatial information while enhancing feature richness.
- Head: The head of YOLOv8 is where the actual detection takes place, utilizing the processed features to predict object classes and bounding boxes. It employs a Detect layer that operates on the fused multi-scale features (P3, P4, P5), directly outputting detection predictions. This design allows YOLOv8 to make predictions at different scales, accommodating small, medium, and large objects within the same framework efficiently.

In our pursuit of refining the perception system for Unmanned Surface Vehicles and ensuring its compatibility with edge devices, we have closely examined the key repeating modules within the YOLOv8 architecture namely, the Conv and C2f modules. These components are pivotal to the model’s functionality, significantly influencing its resource consumption and complexity.

#### 2.2.2. Convolution Module

Standard convolution lies at the heart of many computer vision models, utilizing a linear combination of inputs through a convolutional kernel to extract significant features from images. Mathematically, this operation is expressed as:

(1) $$O\left(i,j\right)=\sum_{m=0}^{K-1}\sum_{n=0}^{K-1}I\left(i+m,j+n\right)\cdot F\left(m,n\right)$$

where O(i,j) denotes the output at position (i,j), *I* represents the input image, *F* is the convolutional filter, and *K* is the size of the filter. This rationale underpins our decision to enhance our model with Depthwise Convolution (DWConv), known for its capacity to significantly reduce computational complexity and resource usage without compromising the model’s ability to accurately detect and analyze visual data. Unlike standard convolution, DWConv operates by separating the convolution process into two distinct steps: depthwise convolution that applies a single filter per input channel and pointwise convolution that combines the outputs of the depthwise convolution across channels.

The depthwise convolution is mathematically represented as follows:

(2) $$O_{c}\left(i,j\right)=\sum_{m=0}^{K-1}\sum_{n=0}^{K-1}I_{c}\left(i+m,j+n\right)\cdot F_{c}\left(m,n\right)$$

where $O_{c}\left(i,j\right)$ is the output for channel *c* at position (i,j), $I_{c}$ is the input for channel *c*, $F_{c}$ is the filter for channel *c*, and *K* is the size of the filter.

Following depthwise convolution, pointwise convolution combines the depthwise outputs into a new set of channels:

(3) $$O\left(i,j\right)=\sum_{c=0}^{C-1}I_{c}\left(i,j\right)\cdot F_{c}$$

where O(i,j) represents the combined output at position (i,j), applying a 1×1 filter across all channels.

**Figure 3:**
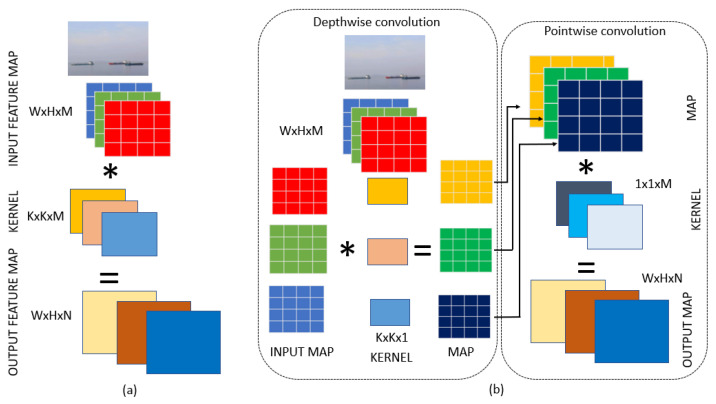
(**a**) Standard convolution, (**b**) depthwise convolution.

Depthwise convolution applies a separate filter to each input channel, reducing computational complexity compared to standard 2D convolutions. Pointwise convolution then uses 1 × 11 × 1 filters to combine these individual channel outputs. This two-step approach significantly lowers the number of calculations and parameters required [47], offering a more efficient way to capture spatial hierarchies in data compared to standard convolution methods, making it especially advantageous for our application. Figure 3 presents a visual presentation between Conventional Convolution and Depthwise Convolution.

#### 2.2.3. C2f Module

In optimizing YOLOv8 for real-time performance on edge devices, we have focused on the C2f module, which merges the Cross Stage Partial (CSP) structure with dual convolution processes. This design divides the feature map into two segments: one for undergoing convolutional changes and the other to preserve initial features. This strategy is key for the network to efficiently integrate both comprehensive and specific data features, aiming for both heightened feature assimilation and computational thriftiness. The process within a C2f module [48] can be conceptualized as follows, although specific operations may vary based on implementation.

Feature Splitting: Given an input feature map *F*, it is split into $F_{1}$ and $F_{2}$.

Convolution Operations:

(4) $$F_{1}^{\prime }=Conv\left(F_{1}\right)$$

where Conv represents convolutional operations applied to part $F_{1}$.

Feature Fusion: The final output $F^{\prime }$ is obtained by concatenating the transformed $F_{1}^{\prime }$ and the bypassed $F_{2}$:

(5) $$F^{\prime }=Concat\left(F_{1}^{\prime },F_{2}\right)$$

**Figure 4:**
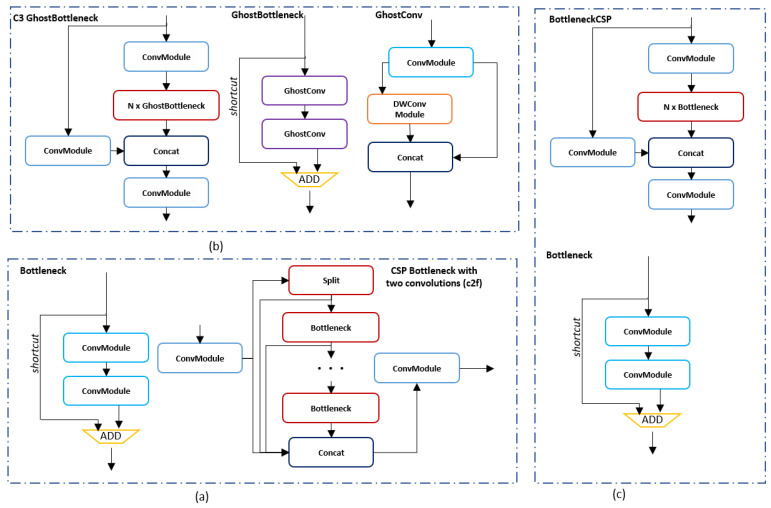
(**a**) BottleneckCSP with two convolutions (c2f), (**b**) C3Ghost, (**c**) BottleneckCSP.

To elevate the YOLOv8 architecture’s C2f module, we meticulously compared the C3Ghost [49] and BottleneckCSP [48] modules, known for their efficient feature processing capabilities. C3Ghost, offering a streamlined alternative to traditional convolutions through ghost convolutions, reduces computational demands while preserving accuracy, making it especially suitable for limited-resource edge devices like the Jetson TX2. Conversely, BottleneckCSP utilizes cross-stage partial connections to enhance feature learning and minimize redundancy, essential for precise object detection. We explored integrating these modules within YOLOv8, through replacement or hybrid approaches with C2f (detailed in a table in the experimental section), aiming to strike an ideal balance between efficiency and performance. Figure 4 provides a visual representation of these modules, illustrating their roles in optimizing the model.

**Figure 5:**
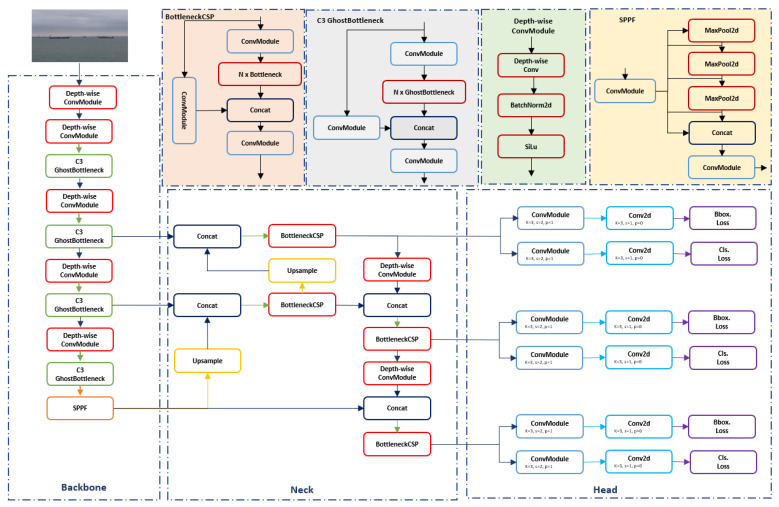
Detailed illustration of our proposed architecture.

Following an exhaustive series of experiments aimed at finding the ideal balance among FPS, energy consumption, and detection precision for our application on the Jetson TX2 equipped USV, our research has culminated in a distinctive architectural configuration. This optimized architecture leverages the strengths of Depthwise Convolution over traditional convolution methods, significantly reducing the overall complexity of the model. In the computationally intensive regions of the backbone, we have integrated the C3Ghost module, known for its efficiency in feature extraction without substantial computational load. Additionally, for the neck section of the architecture, where feature integration is critical, we substituted the standard C2f module with the BottleneckCSP module, renowned for its effectiveness in minimizing feature redundancy while enhancing the model’s learning capability. A detailed illustration of our refined architecture is featured in the subsequent Figure 5.

While detection is vital, maintaining continuous observation of identified targets requires an efficient tracking system, which we detail in the following section.

#### 2.2.4. Tracking System

The Tracking System module is a key component for maintaining continuous surveillance of detected objects as they move through the USV’s field of vision. This capability is essential for mission-critical applications such as maritime monitoring, where real-time tracking ensures that targets of interest are not lost during navigation. The module’s role in sustaining focus on identified objects significantly enhances the USV’s operational effectiveness and reliability.

Within the domain of autonomous USV surveillance, the selection of an efficient and effective tracking algorithm is paramount. Our system leverages the established Centroid Tracker, chosen for its balance of reliability and computational efficiency. This section outlines the rationale behind adopting the Centroid Tracker over other prevalent tracking algorithms for our USV, operated via a Jetson TX2 onboard computer. The decision is underscored by the Centroid Tracker’s suitability for real-time operations in a resource-constrained environment, where power consumption and processing capabilities are critical considerations. To justify our choice, we present a comparison of several leading tracking algorithms, evaluating them across dimensions crucial to autonomous USV operations. These dimensions include algorithmic complexity, reliability in varied operational scenarios, computational overhead, and energy consumption on the Jetson TX2 platform. Table 1 seeks to highlight the Centroid Tracker’s standout features, especially its simplicity and lower energy requirements, essential for extending USV missions’ duration.

Upon successfully loading the video feed into our tracking system, the mechanism initiates a continuous loop of frame analysis. The system’s operation ceases under two specific conditions: either when it encounters a frame that cannot be processed or when the pilot actively deactivates the tracking functionality. At the heart of this sophisticated operation lies the Centroid Tracker algorithm. This method is characterized by its lightweight yet effective strategy for tracking objects across sequential video frames. It begins with the detection phase, where objects are identified, and bounding boxes are delineated. For each detected object, the Centroid Tracker calculates the centroid (the geometric center) employing a straightforward yet potent formula:

(6) $$C=\left(\frac{x_{1}+x_{2}}{2},\frac{y_{1}+y_{2}}{2}\right)$$

where *C* represents the centroid of a bounding box with corners at $\left(x_{1},y_{1}\right)$ and $\left(x_{2},y_{2}\right)$.

This simplicity belies the algorithm’s robust capability to seamlessly transition from passive observation to active tracking upon pilot command. Initially dormant, tracking springs into action with the pilot’s selection, initializing with the first detected object and its calculated centroid. This one-time setup phase lays the groundwork for continuous, dynamic tracking. Once operational, the system perpetually updates the tracked object’s position, drawing from the core principle of minimizing the Euclidean distance between object centroids across frames:

(7) $$D=\sqrt{{\left(x_{i}-x_{j}\right)}^{2}+{\left(y_{i}-y_{j}\right)}^{2}}$$

This mathematical procedure guarantees that objects are reliably followed based on their centroid locations. The output from the Centroid Tracker is then relayed to the control block, enabling the physical tracking of objects by the USV through sophisticated regulation and stabilization algorithms.

To support these computational processes over extended periods, an effective energy management strategy is essential. The next section discusses how our energy management module optimizes power consumption to sustain long-duration missions

### 2.3. Energy Management

The Energy Management module is essential for ensuring the sustainability of autonomous USV missions by efficiently regulating the power usage of onboard systems. This module’s capability to dynamically adjust between various states of computational activity optimizes energy consumption, thereby extending mission duration and enhancing the reliability of the USV in prolonged operations.

In autonomous maritime operations, efficient energy management of onboard systems like the Jetson TX2 is pivotal. Our strategy focuses on dynamically toggling between computational states ranging from a sleep state to active surveillance modes. This adaptability ensures the Jetson TX2’s energy consumption is optimized for the task at hand, balancing between full algorithmic engagement and selective processing based on immediate needs. Our analysis reveals a nuanced energy landscape: full activation of detection, identification, and tracking processes consumes between 8626 mJ to 9423 mJ, while a detection-only mode significantly lowers this range to 5459 mJ to 6464 mJ. Surprisingly, a tracking-focused scenario averages a consumption of 7200 mJ, offering a middle ground. These data highlight the value of strategic algorithm activation in extending USV mission durations and conserving energy. By aligning computational efforts with situational demands, we ensure not only the sustainability of operations but also the efficient use of the Jetson TX2’s capabilities in enhancing autonomous maritime surveillance.

## 3. Hardware and Software Implementation

### 3.1. Hardware Platform

To evaluate the models on a specified hardware setup, we employed a high-performance computing system equipped with a Quadro RTX 5000 Nvidia GPU, 32 GB of RAM, and an Intel Xeon CPU operating at 3.60 GHz with eight cores. The model training and evaluation were performed on this configuration.

For the experiments, the system ran Ubuntu 20.04 LTS, leveraging CUDA 12.3.0 to facilitate accelerated training on the Quadro RTX 5000. In contrast, the Jetson TX2 was configured with Ubuntu 18.04 and CUDA 10.2, utilizing OpenCV for image processing tasks. The training utilized PyTorch, with versions tailored to each device: PyTorch 2.1.2 and OpenCV 4.7.0 for the RTX 5000, and PyTorch version 1.7.0 along with OpenCV version 4.7.0 for the Jetson TX2.

### 3.2. Sea Ship Dataset

To construct a robust classifier, it is imperative to curate a dataset encompassing training images that exhibit a diverse array of conditions. These conditions may encompass images with miscellaneous objects, varying lighting conditions, diverse backgrounds, partially obscured regions of interest, and instances of overlap. The Sea Ship dataset [57] serves as a valuable resource for validating these conditions, as it comprises images captured by cameras within a real-world video surveillance system.

This system comprises 156 cameras strategically positioned across 50 distinct locations along the northwest border of Hengqin Island, collectively covering an expanse of 53 square kilometers of coastal regions. Each location is equipped with three cameras, including one low-light HD camera and two bolt-type HD cameras. A total of 7000 images were extracted from the Sea Ship dataset for utilization in this study, with the dataset being categorized into several classes. Table 2 delineates the different classes along with the corresponding number of images in each.

### 3.3. Performance Metrics

In this study, we conducted a comprehensive evaluation of object detection models using a diverse set of metrics aimed at capturing various aspects of model quality and efficiency. To assess the precision and recall capabilities of the models, we computed precision and recall metrics, providing insights into the models’ ability to accurately detect ships under different conditions. Furthermore, to achieve a thorough assessment of model generalization capability, we computed mean Average Precision (mAP) at different thresholds, including mAP0.5 and mAP0.95. These metrics allowed us to compare model quality based on different tolerance levels for false positives and false negatives, offering an overall view of model performance in real-world scenarios.

Concurrently, we evaluated the complexity and computational efficiency of the models by analyzing the number of layers, parameters, and gradients. These measures provided insights into the models’ complexity and their ability to learn rich ship representations in images. Additionally, we calculated the number of Floating Point Operations Per Second (GFLOPS) to assess the computational workload of the models, providing indications of their performance on resource-constrained platforms such as the Jetson TX2. Training time was also recorded to evaluate the temporal efficiency of model training.

Regarding performance evaluation on the Jetson TX2, we also utilized two key indicators: Frames Per Second (FPS) and energy consumption. FPS offered insights into the inference speed of the models under real-world conditions, while energy consumption allowed us to assess the models’ energy efficiency, a key aspect in embedded applications. These indicators enabled us to select models best suited to real-time performance constraints and energy efficiency in an embedded context.

## 4. Results and Discussion

In this section, we present the outcomes of training and testing various configurations of the YOLOv8 object detection model. These models were trained using an identical dataset and under uniform conditions, with training images sized at 640 × 640 pixels. To evaluate the models’ effectiveness, we utilized real-time videos that simulated the actual conditions of our intended application, incorporating factors such as waves, fog, and reflections. These videos included images not originally part of the dataset. The training configurations employed are elaborated in Table 3. We analyze different performance indicators to assess the efficiency of each model. The models were compared based on several evaluation metrics, including precision, recall, Map0.5, Map0.95, layers, parameters, gradients, GFLOPS, and training time. The results are presented in Table 4.

Following Table 5, which shows the detailed examination of training outcomes, our analysis zeroes in on the architectural variations across the YOLOv8 configurations. These variations hinge on the strategic use of Convolutional layers, Depthwise Convolutional layers, the innovative c2f and C3Ghost modules, and the BottleneckCSP architecture. Each configuration was designed to balance computational efficiency with the capability to accurately detect objects in challenging environmental conditions, such as waves, fog, and reflections, which are typical in maritime surveillance scenarios.

**Figure 6:**
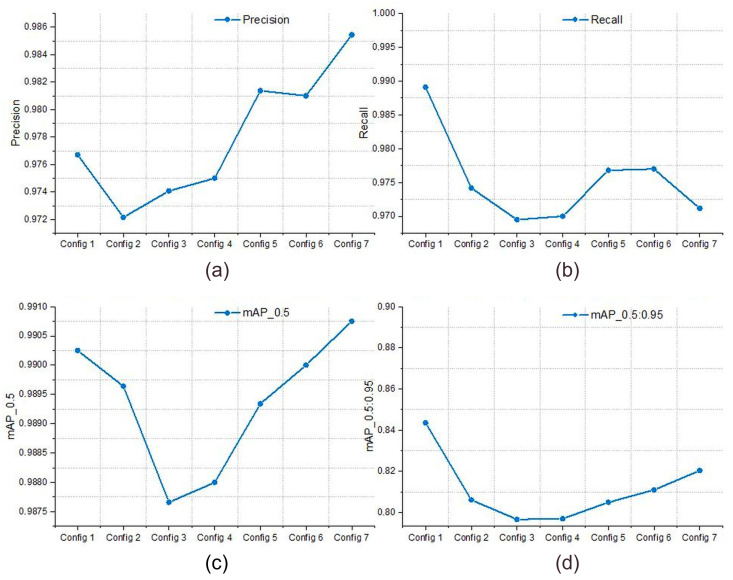
Performance metrics of custom YOLO architectures: (**a**) Precision metrics, (**b**) Recall metrics, (**c**) mAP0.5, and (**d**) mAP0.95 for various YOLO models.

Summarizing the analysis, the examination of various model configurations, as detailed in Table 4 and illustrated in Graph in Figure 6, highlights the models’ performance in object detection accuracy, with a particular emphasis on the detection of sea ships. Among the various configurations tested, Configuration 7 stands out due to its exceptional precision (0.98545), indicating its superior ability to accurately identify sea ships with minimal false positives. This configuration also shows robust performance in terms of Recall (0.97116) and maintains high mAP scores at both IoU (Intersection over Union) thresholds of 0.5 and 0.95 (0.99075 and 0.8204, respectively), suggesting its effectiveness in recognizing a high percentage of sea ship objects accurately across various sizes and shapes. Delving deeper into the architectural components of Configuration 7, as depicted in Table 5, it is noteworthy that the inclusion of Depthwise Convolutional layers, combined with Convolutional layers and the selective use of both C3Ghost modules and BottleneckCSP structures, plays a key role in its leading performance. These components are known for their efficiency in processing and feature extraction capabilities, which are vital for capturing the nuanced details required for precise object detection in maritime environments.

In comparison to Configuration 1, which exhibits efficiency with the best Recall (0.98909), Configuration 7 emerges as the superior choice due to its overall balance. Configuration 7 achieves an optimal equilibrium between computational efficiency and precision, boasting a GFLOPS of 5.8 and completing training in just 2.91 h. This balance is pivotal for practical scenarios where achieving high accuracy without excessive resource consumption is essential. Additionally, Configuration 7’s architectural innovations and balanced performance across all metrics make it a particularly effective solution for maritime object detection tasks.

**Figure 7:**
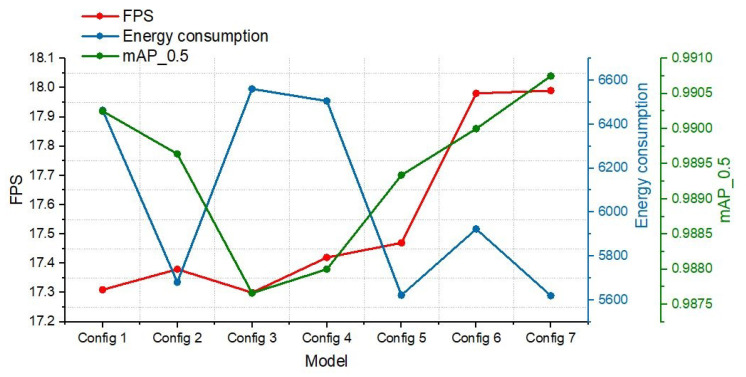
Correlation of FPS, energy consumption, and Map0.5 across YOLO model configurations.

Table 6 outlines various YOLO model configurations with their respective performance metrics including FPS, energy consumption, and detection precision (Map0.5), while the graph in Figure 7 depicts the correlation among these metrics for each configuration tested on the TX2 platform. This graph highlights the trade-offs between FPS, energy consumption, and detection precision (mAP0.5), showcasing the strengths and compromises of each configuration. Notably, Configuration 7 demonstrates its superiority by achieving an FPS of 17.99 and maintaining the lowest energy consumption at 5619.54 J, coupled with the highest mAP0.5 of 0.99075. This indicates that Configuration 7 offers an optimal balance between high detection precision and efficient energy usage, making it particularly well-suited for real-time maritime surveillance on resource-constrained platforms like the Jetson TX2. In contrast, other configurations, such as Configuration 1, show strong recall but fall behind in terms of energy efficiency and overall precision. The results underscore the importance of selecting a model configuration that achieves a harmonious balance of accuracy, speed, and energy consumption for practical deployment in maritime environments.

Our analysis on Jetson TX2 for ship detection with YOLOv8 configurations highlighted the trade-offs between detection accuracy, energy efficiency, and real-time processing. Higher FPS rates often increased energy consumption, posing challenges for sustained usage. However, not all high-energy-consuming models guaranteed superior detection accuracy, emphasizing the importance of balancing computational resources and precision. The additional performance metrics emphasize Configuration 7’s exceptional capabilities in object detection tasks, especially within maritime applications. With the highest frames per second rate at 17.99, this setup showcases unparalleled real-time processing abilities, essential for applications that require quick and efficient maritime monitoring and object detection. Although its energy consumption is the lowest, standing at 5619.54 joules, it is deemed a reasonable trade-off considering its outstanding efficiency and the highest mAP at 0.5 IoU of 0.99075 among the configurations evaluated. In the context of balancing energy consumption, FPS performance, and precision, Configuration emerges as the superior choice. This balance clearly positions it as the winner in the triangle of energy consumption, FPS, and accuracy, demonstrating its aptitude for delivering high performance in challenging maritime environments with moderate energy requirements.

Building on comparisons with the existing literature, all of which utilize the same dataset and focus on edge devices, our analysis significantly highlights the remarkable performance of Configuration 7 on the Jetson Tx2 platform within the landscape of edge computing solutions, as presented in Table 6 and Table 7. This specific setup demonstrates a balance between high detection accuracy, as evidenced by its mAP of 0.99075, and a reasonable energy consumption of 5.61954 joules. Such results position our configuration as a strong competitor against a variety of computing platforms traditionally employed in similar scenarios, further solidifying its suitability for energy-sensitive, high-accuracy detection tasks in edge environments.

**Figure 8:**
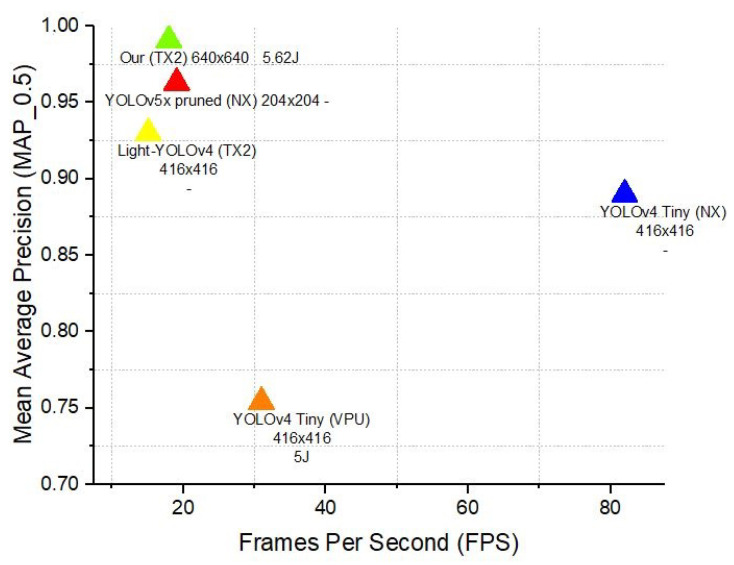
Comparison of object detection methods on different hardware platforms.

**Figure 9:**
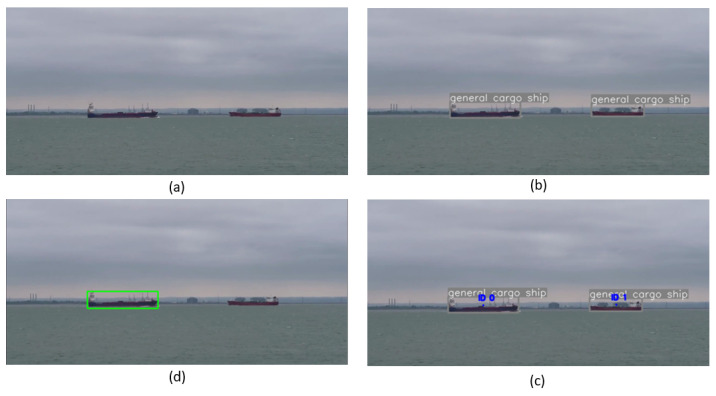
Comprehensive visualization of the detection and tracking process using our model. (**a**) Original image without detection, showcasing the real maritime environment. (**b**) Result of our detection model. (**c**) Detection and identification results. (**d**) Outcome of our tracking algorithm, demonstrating the continuous monitoring of identified objects.

**Figure 10:**
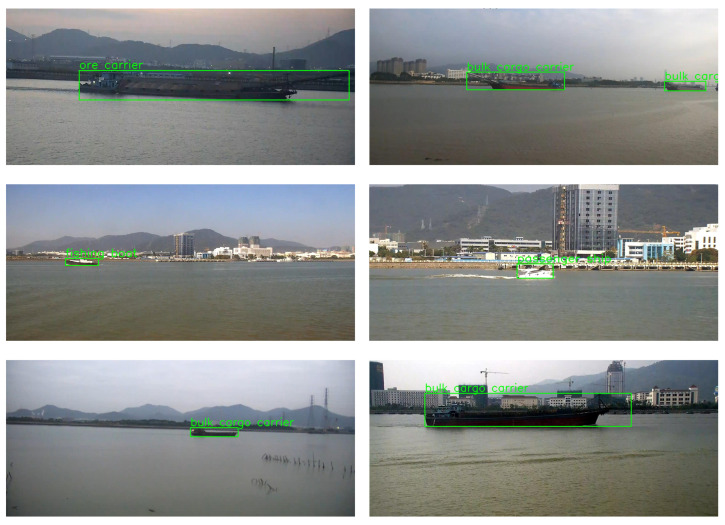
Model performance demonstration under adverse weather conditions (fog, wind, and sea surface reflections.

Compared to models on other platforms like the Jetson Xavier NX and the MOVIDIUS MYRIAD X VPU, our Configuration 7 demonstrates superior accuracy. For instance, models like YOLOv4 Tiny and YOLOv5x pruned, running on the Jetson Xavier NX, achieve notable FPS rates but lag behind in mAP levels, especially at 640 × 640 resolution. Our model excels in both speed and accuracy, challenging the usual trade-off between the two in object detection systems. Furthermore, Configuration 7’s energy efficiency remains notably competitive, even when compared to devices like the Movidus MyRiad X vpu, renowned for their low power consumption. Our configuration demonstrates that achieving high accuracy does not necessarily lead to increased energy consumption, effectively striking a balance to meet the demands of edge computing applications. Additionally, our analysis reveals Configuration 7’s exceptional efficiency on the same platform, outperforming Light-YOLOv4 in both accuracy and FPS. This comparison underscores Configuration 7’s versatility across various hardware platforms, as depicted in Figure 8. Our methodology showed outstanding performance in both mAP and FPS metrics, securing the highest mAP and competitive FPS rate among assessed models. This highlights the efficacy and operational efficiency of our method, making it highly viable for object detection in USV applications. Additionally, Figure 9 demonstrates our model deployed on the NVIDIA Jetson TX2 edge device onboard our USV, illustrating its practical application and effectiveness in real-world maritime surveillance. Furthermore, Figure 10 provides a demonstration of the model’s performance under challenging weather conditions, including fog, wind, and reflections on the sea surface. These real-world examples underscore the practical value of our model in handling adverse conditions and managing false detections effectively.

## 5. Conclusions

This study presents a breakthrough in maritime surveillance with an AI-powered detection and tracking system optimized for USVs. A highlight is the development of a USV architecture and a perception module tuned for high-performance object detection while maintaining energy efficiency. Implementation on the NVIDIA Jetson TX2 sets a new benchmark in detection accuracy and energy optimization, serving as a model for edge computing in real-time maritime surveillance. Through comprehensive analysis, our configuration achieves an mAP of 0.99 and operates at 17.99 FPS, with an energy consumption of just 5.61 joules. This balanced approach validates the potential of AI integration in maritime surveillance, promising enhancements in safety, security, and environmental monitoring.

## Figures and Tables

**Table 1:** Comparison of tracking algorithms for USV autonomous surveillance.

Algorithm	Complexity	Reliability	Computational Overhead	Power Consumption
Centroid Tracker [53]	Low	High	Low	Low
KCF [54]	Medium	High	Medium	Medium
SORT [55]	Low	Medium	Low	Low-Medium
DeepSORT [56]	High	High	High	High

**Table 2:** Number of images in each class in the dataset.

Classes	Number of Images
Ore Carrier	2200
Bulk Cargo Carrier	1953
Container Ship	1505
General Cargo Ship	901
Fishing Boat	2190
Passenger Ship	474
Total	9223

**Table 3:** Parameters used for training the YOLO object detection model.

Parameters	Value
Batch size	16
Momentum	0.73375
Learning rate	0.00269
Number of iterations	250 epochs
Fixed image size	640 × 640
Weight decay	0.00015

**Table 4:** Description of the various configurations with their associated metrics.

No.	Precision	Recall	Map0.5	Map.5:.9	Layers	Param.	Grad.	GFLOPS	Train (h)
1	0.97671	0.98909	0.99025	0.84351	225	3,012,018	3,012,002	8.2	2.145
2	0.97215	0.97418	0.98964	0.80610	379	1,999,734	1,999,718	5.7	2.887
3	0.97407	0.96949	0.98766	0.79669	257	2,955,474	2,955,458	8.1	3.181
4	0.97500	0.97000	0.98800	0.79700	237	3,045,465	3,045,449	8.3	2.960
5	0.98138	0.97679	0.98934	0.80500	343	1,597,758	1,597,742	5.3	2.790
6	0.98100	0.97700	0.99000	0.81100	257	2,385,522	2,385,506	6.9	2.878
7	0.98545	0.97116	0.99075	0.82040	236	1,911,642	1,916,442	5.8	2.910

**Table 5:** Description of the different configurations.

No.	Conv	DWConv	c2f	C3Ghost	BCSP
1	Yes	No	Yes	No	No
2	Yes	No	No	Yes	No
3	Yes	No	No	No	Yes
4	No	Yes	Yes	No	No
5	No	Yes	No	Yes	No
6	No	Yes	No	No	Yes
7	No	Yes	No	Yes	Yes

**Table 6:** Comparative performance metrics of different configurations.

Config	FPS	Energy (J)	Map0.5
1	17.31	6464.04	0.99025
2	17.38	5680.80	0.98964
3	17.30	6561.40	0.98766
4	17.42	6506.26	0.98800
5	17.47	5621.90	0.98934
6	17.981	5922.86	0.99000
7	17.99	5619.54	0.99075

**Table 7:** Performance and energy consumption of different models on various platforms.

Model	FPS	Map_0.5	Resolution	Energy (J)	Platform
YOLOv4 Tiny [50]	82	0.89	416 × 416	N/A	Jetson Xavier NX
YOLOv5x pruned [51]	19.09	0.963	204 × 204	N/A	Jetson Xavier NX
YOLOv4 Tiny [36]	31	0.7541	416 × 416	5	MOVIDIUS MYRIAD X VPU
Light-YOLOv4 [34]	15.12	0.930	416 × 416	N/A	NVIDIA Jetson TX2
Our Enhanced YOLOv8	17.95	0.99075	640 × 640	5.61954	Jetson TX2

## Data Availability

Data are contained within the article.
